# Election forensics: Using machine learning and synthetic data for possible election anomaly detection

**DOI:** 10.1371/journal.pone.0223950

**Published:** 2019-10-31

**Authors:** Mali Zhang, R. Michael Alvarez, Ines Levin

**Affiliations:** 1 Division of Humanities and Social Sciences, California Institute of Technology, Pasadena, CA, United States of America; 2 Department of Political Science, University of California, Irvine, CA, United States of America; Universidade Estadual de Maringa, BRAZIL

## Abstract

Assuring election integrity is essential for the legitimacy of elected representative democratic government. Until recently, other than in-person election observation, there have been few quantitative methods for determining the integrity of a democratic election. Here we present a machine learning methodology for identifying polling places at risk of election fraud and estimating the extent of potential electoral manipulation, using synthetic training data. We apply this methodology to *mesa*-level data from Argentina’s 2015 national elections.

## Introduction

Assuring that an election was run in a free and fair manner has been the focus of a significant body of research. Much of that research, especially efforts to document the integrity of elections outside the United States, has utilized direct in-person election observation methods [1, 2]. In-person election observation can be an effective tool for detecting and deterring election fraud [3]; however it is costly to implement, and in a large national election where there may be tens or hundreds of thousands of polling places requiring observation, it can be difficult or impossible to provide sufficient coverage for assurance that the election was conducted freely and fairly.

The desire for a less-costly, quantitative, and broad coverage means of detecting potential election fraud has led researchers to propose a number of quantitative approaches for detecting irregularities using different methods for anomaly detection [4]. Some have advocated for the examination of the distributions of elections data (especially turnout), arguing that oddly distributed elections data can often indicate problems in the administration of an election [5]. Others have studied the use of “digit-tests”, which look for anomalies in the distribution of digits in multi-digit elections data [6–8], but see [9]. Researchers have also used “flow of votes” analyses, parametric statistical models, or statistical tests to look for anomalies in elections data [10–17].

These various approaches for detecting potential election fraud all suffer from different methodological problems [18]. These relatively simple forensics tools can be difficult to use at the scale of a large national election: they do not necessarily take into consideration information from past elections, they do not consider *a priori* knowledge about the forms that election manipulation may take in a particular context, and may fail to detect many forms of potential fraud.

These methodological issues have led some scholars to advocate the use of machine learning approaches for detecting potential election fraud [18–21]. There are many advantages of using machine learning approaches for trying to detect potential fraud. In particular, they can easily make use of large amounts of election data, finding anomalous observations, and can do so using methodologies that make minimal parametric and distributional assumptions. Additionally, machine learning allows us to avoid the reliance on single models, and instead we can use ensembles of models to improve our ability to find election anomalies. Finally, as we argue in this paper, we can incorporate into our machine learning models the knowledge that social scientists have accumulated about election regularities—and potential election fraud—to increase the likelihood that our models detect the types of anomalies in elections data that may be the result of manipulation or error.

Our approach differs from those in previous studies. While we use a supervised machine learning ensemble approach (Random Forest), we lack a priori labeling of voting precincts for whether they may have been affected by manipulation—and the form and extent of that electoral manipulation. Thus, we develop a relatively naïve hierarchical model, that we use to estimate what a “clean” election might look like across all of the voting precincts in the election we study, which forms our “clean” synthetic training data. Based on our substantive knowledge of the election, and on past studies of election fraud, we focus on two forms of manipulation: ballot-box stuffing and vote stealing. We then use our a priori and theory-driven knowledge about the potential forms and extent of potential election fraud to generate synthetic examples of voting precincts exposed to manipulation. We fit the model using cases labeled as ‘clean’ and ‘at risk’ in the synthetic training data, then apply that trained model to national data from the 2015 national elections in Argentina. With this method we are able to locate polling precincts resembling training examples susceptible to manipulation or error, and to estimate the extent of this potential electoral manipulation. This approach is discussed in detail below, where we also provide a variety of validation analyses.

## Data and methods

### Data

We use data from the first round of the 2015 presidential election in Argentina at the polling station (*mesa*) level [22]. This dataset contains information about the number of votes received by the six parties in the national election, the number of invalid and blank votes, and the number of eligible voters. The six parties include three larger parties, Frente para la Victoria (FPV), Cambiemos, Unidos por una Nueva Alternativa (UNA), and three smaller parties, Progresistas, Compromiso Federal, and Frente de Izquierda y de Los Trabajadores. Since the smaller parties received zero votes in many precincts, we combine their votes into votes for “other” parties to facilitate modeling. We supplement this data by incorporating results from the previous presidential election and demographic information (such as home ownership, gender ratio, illiteracy, and age) at the department level. We focus on rows from the electoral data corresponding to *mesas* where votes were cast for the larger parties and the combined “other” party. We remove *mesas* with less than 100 total ballots cast, as these very small *mesas* have highly variable turnout and vote share results. After removing these cases, we have 90,012 *mesas* from 24 provinces (96% of the original sample size). A summary of the voting results from these *mesas* is shown in Table 1. For a full description of data sources, see the replication materials [23].

**Table 1:** Summary statistics of turnout and vote shares.

	Min.	Median	Mean	Max.
Electorate	110	350	340	390
Turnout	0.30	0.82	0.81	1.00
Vote Share				
FPV	0.01	0.27	0.29	0.88
Cambiemos	0.00	0.26	0.27	0.80
UNA	0.00	0.17	0.17	0.55
Other	0.00	0.05	0.06	0.76
Residual	0.00	0.03	0.03	0.66

This table, from top to bottom row, summarizes the distributions of the size of electoral population, turnout, and vote shares (measured as a proportion of the electorate) received by various parties across all 90,012 *mesas*.

We focus on the 2015 election as it took place in the context of widespread distrust in the integrity of the electoral process. A recent scholarly publication presented evidence suggestive of election irregularities in the previous presidential election [24]. Uncertainty about election integrity remained an issue in 2015. In September 2015, news reports circulated about allegations of election fraud in a gubernatorial election in Tucuman [25]. The general election, which took place only a month after these events, was set against a background of allegations of election irregularities and doubts about ballot secrecy [26]. These concerns make this election an ideal case for exploring possible anomalies in election returns.

### Synthetic data generation

We generate synthetic clean and at-risk data to train a supervised classification model that can be used on the actual election data to classify *mesas* into clean or at-risk categories. We first generate clean synthetic data using a mixed effects regression. We use the vote share of each party as the dependent variable, and incorporate demographic variables as well as whether FPV won a majority of votes in 2011 as fixed effects, while allowing the intercepts to vary by department. We use this model to predict the expected clean vote share for each party, given their demographics and results from 2011.

One concern is that the actual vote shares on which we train the multilevel model may themselves be tainted by some type of manipulation, and hence the model predictions may not accurately reflect an entirely “clean” distribution. However, by using potentially manipulated data as the “clean” baseline for the model, it tempers the overall distribution of our predictor variables, which makes extreme values less unlikely. As our models seek anomalous behaviors in the turnout and vote shares, this tempering means that our model is more conservative in detecting voting locations that may be at risk for manipulation or error.

Next, we alter the clean synthetic data in two ways that fall into one of the two hypothetical fraudulent behaviors: (1) vote stealing (VS), where agents from one of the major parties steal votes from other parties by transferring some of these votes to the perpetrating party and destroying the rest, and (2) ballot box stuffing (BBS), where party agents artificially inflate the votes for their party. Here we focus on the FPV, the incumbent Peronist party, for two reasons. First, a previous study found that FPV monitors may have interfered with election results in areas covered by their party monitors in past elections [24]. Second, the other two major parties in contention did not have strong capacity or incentive to commit potential fraud. Cambiemos was a new party in the 2015 election and hence did not have comparable ability to access and cover large areas with its party monitors, whereas UNA did not have a substantial chance of winning against FPV and thus had no incentive to commit these two types of possible fraud.

**Fig 1:**
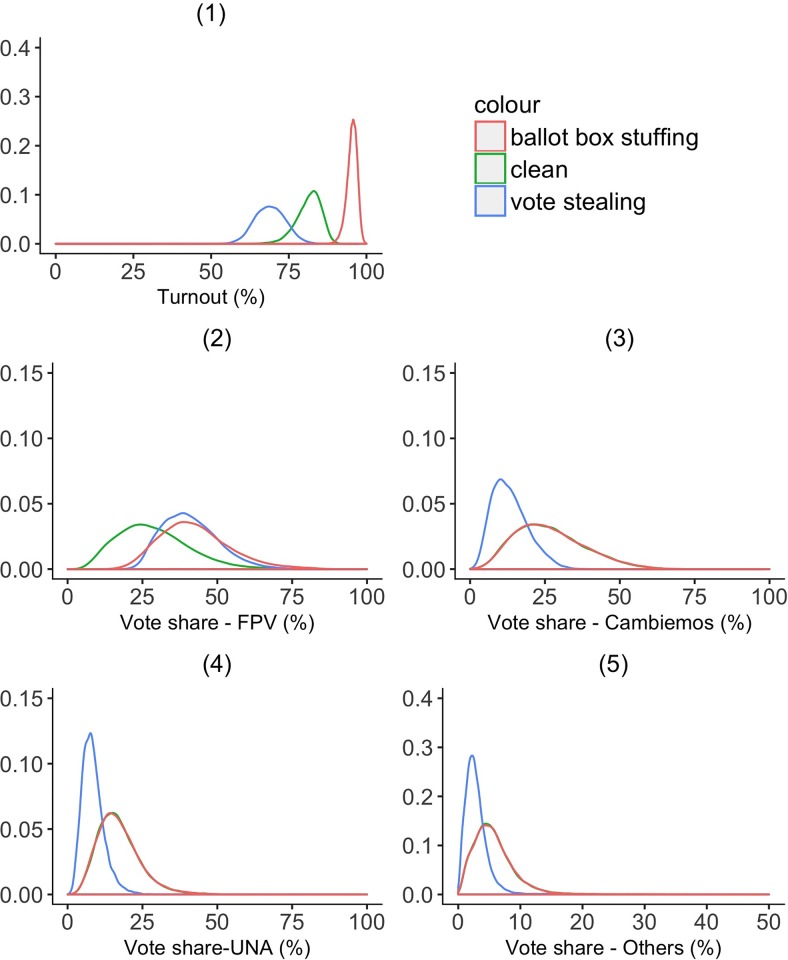
Distribution of electoral variables in synthetic training data. *N* = 86,815; *N*_*Clean*_ = 29,093; *N*_*BBS*_ = 28,999; *N*_*VS*_ = 28,723. These figures show turnout and the vote share of different parties across *mesas* that are simulated to be either: (1) at risk of ballot box stuffing (shown by the red line), (2) clean (shown by the green line), or (3) at risk of vote stealing (shown by the blue line).

We generate *at-risk* synthetic examples as follows. We choose two separate parameters that dictate the probability of any *mesa* being tainted by VS and BBS, and two additional parameters that determine the extent of VS or BBS in tainted *mesas*. The extent of VS is given by the proportion of votes from other parties being converted to FPV votes. The extent of BBS is given by the proportion of abstention votes counted as FPV votes. We then use a random process to choose which at-risk *mesas* face which type of potential fraud, and the extent of potential fraud in affected mesas. As the next step in our synthetic data generation, we subtract the potential stolen votes from the other parties and give a portion of them to FPV, and add additional votes to FPV to simulate ballot box stuffing. On average, synthetic *mesas* at risk of VS show relatively small turnout rates, whereas those at risk of BBS have high turnout rates. Both types of simulated manipulation show higher vote shares for the FPV. Distributions of turnout and the vote share of each party are shown in Fig 1. As can be seen in panel (1) of Fig 1, the simulated turnout is lowest with VS, and highest with BBS. Panel (2) shows that both VS and BBS would contribute positively to the vote share of FPV. The remaining panels (3-5) show that while BBS does not have a substantial impact on the vote share of other parties, VS does drastically lower other parties’ vote shares.

### Modeling

We then use the labeled synthetic data to train a model that can be used on actual election data to see whether there is evidence suggesting that a voting precinct is at risk of VS or BBS. The outcome variable of the model has three classes: clean, risk of VS, or risk of BBS. The predictors are turnout and the vote shares of each party. We use Random Forest to train this model [27]; Random Forest is an ensemble supervised machine learning approach, which our previous work has shown works well for detecting potential election fraud [18].

To test the performance of our model, we divide the synthetic data into 10 folds, train the model on 9 folds, and examine its performance on the remaining one fold of data saved for testing. We average the prediction accuracy for the 10 tests and show the performance in Table 2. As Table 2 suggests, the model does a highly accurate job predicting whether a synthetic *mesa* is clean or at risk of manipulation, correctly predicting 97% of the clean synthetic examples.

**Table 2:** Random Forest model performance on synthetic training data.

Actual	Prediction		
	Clean	BBS risk	VS risk
Clean	0.965	0.002	0.032
BBS risk	0.004	0.996	0.000
VS risk	0.010	0.000	0.990

This table presents prediction accuracy of a Random Forest model trained using 90% of the synthetic data on the remaining 10% of the synthetic data. The proportions in this table are the average of 10 models, where we divide the data into 10 folds, train a Random Forest model on 9 folds, and predict the outcome in the remaining one fold.

**Fig 2:**
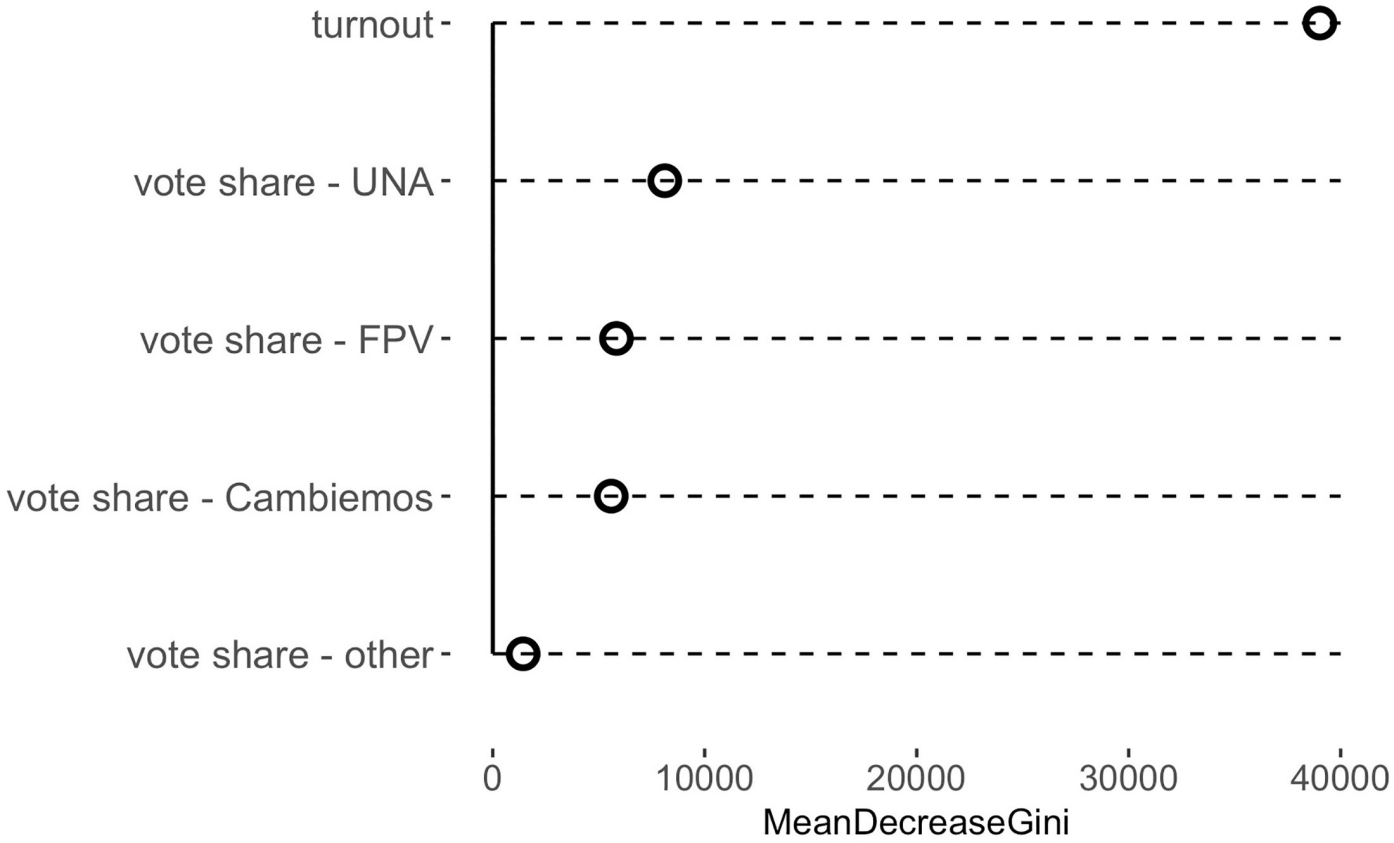
Importance of predictors. This figure shows the importance of variables used in the Random Forest model trained on synthetic data. The importance here is measured by the average decrease in the Gini index across decision trees upon permutation of the values of each predictive feature.

We next train a Random Forest model on the full set of synthetic data to utilize all available information. Fig 2 shows permutation-based measures of the importance of each variable in this Random Forest model for predicting at-risk status. Turnout is by far the most important predictor for fraud, followed by the vote shares of the three largest parties. By construction, the vote share of other smaller parties does not contribute much to the overall fit. Finally, we apply this model to the actual data and classify each *mesa* into one of the three categories: clean, at risk of BBS, or at risk of VS.

## Results

We first present the prediction results using a set of discretionary parameters. We show the proportion of clean and at risk *mesas* in the prediction, as well as the distribution of turnout and vote shares. Then we evaluate the sensitivity of results to changes in key parameters used to generate the synthetic data—the probability an at-risk *mesa* is tainted with potential fraud or error and the extent of the manipulation in at risk *mesas*.

Table 3 summarizes the proportion of *mesas* that are predicted to be clean and at risk in the actual data, using the Random Forest model trained on randomly generated synthetic data drawn from hypothetical clean and at-risk distributions. In generating the synthetic data, we initially assume that 1/3 of at-risk mesas are affected by BBS and VS, respectively, and that these two forms of fraud are exclusive events. We also assume that, on average, 3/4 of voter abstention in *mesas* at risk of BBS is counted as votes for the incumbent, and that 1/2 of votes cast for opposition parties in *mesas* at risk of VS are counted as votes for the incumbent. Setting these last two parameters at large levels helps limit the chance that clean *mesas* closely resembling the at-risk data will by chance be classified as at-risk. When simulation parameters are held at these baseline levels, 86.3% of *mesas* are predicted to be clean in the actual data. In total, 13.7% of *mesas* are classified as at-risk; 12.3% are found at risk of BBS and only 1.4% at risk of VS.

**Table 3:** Percentage of clean and at risk *mesas* in real data.

	Clean	At risk	BBS risk	VS risk
All *mesas*	86.30	13.70	12.32	1.38

This table shows the predicted percentage of at risk *mesas* (either through possible ballot box stuffing or vote stealing), using the Random Forest model trained on synthetic data. Key parameters used in generating the synthetic training data: probability for any *mesa* to be subject to BBS = 1/3, probability for any *mesa* to be subject to VS = 1/3, proportion of votes stolen = 1/2, proportion of abstention votes being stuffed = 3/4.

**Fig 3:**
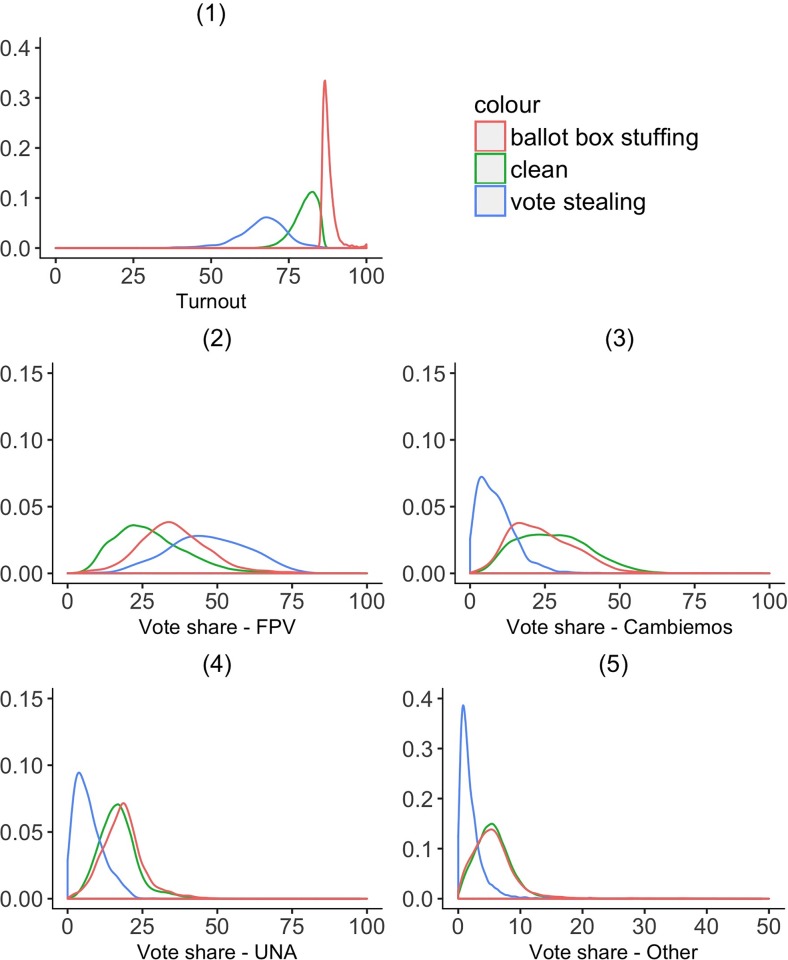
Distribution of electoral variables in actual data by predicted risk. *N* = 86,815; *N*_*Clean*_ = 76,604; *N*_*BBS*_ = 9,014; *N*_*VS*_ = 1,197. These figures show turnout and vote share of different parties across *mesas* that are predicted by the Random Forest model to be either: (1) at risk of ballot box stuffing (shown by the red line), (2) clean (shown by the green line), or (3) at risk of vote stealing (shown by the blue line).

Fig 3 presents the distributions of key metrics, turnout and shares for different parties, across different categories of *mesas*. Similar to the synthetic data, *mesas* at risk of BBS have much higher turnout, while turnout is the lowest in *mesas* at risk of VS. While VS and BBS have somewhat similar effects on the vote share of the FPV in the synthetic data, *mesas* at risk of VS show much stronger support for FPV than *mesas* at risk of BBS. Unsurprisingly, the risk of VS is significantly associated with lower vote shares for all other parties, whereas the risk of BBS is associated with decreased support for Cambiemos more than the other parties. Overall, the distributions of turnout and vote shares in the actual data using model predicted categories are consistent with the distributions in the synthetic data.

**Fig 4:**
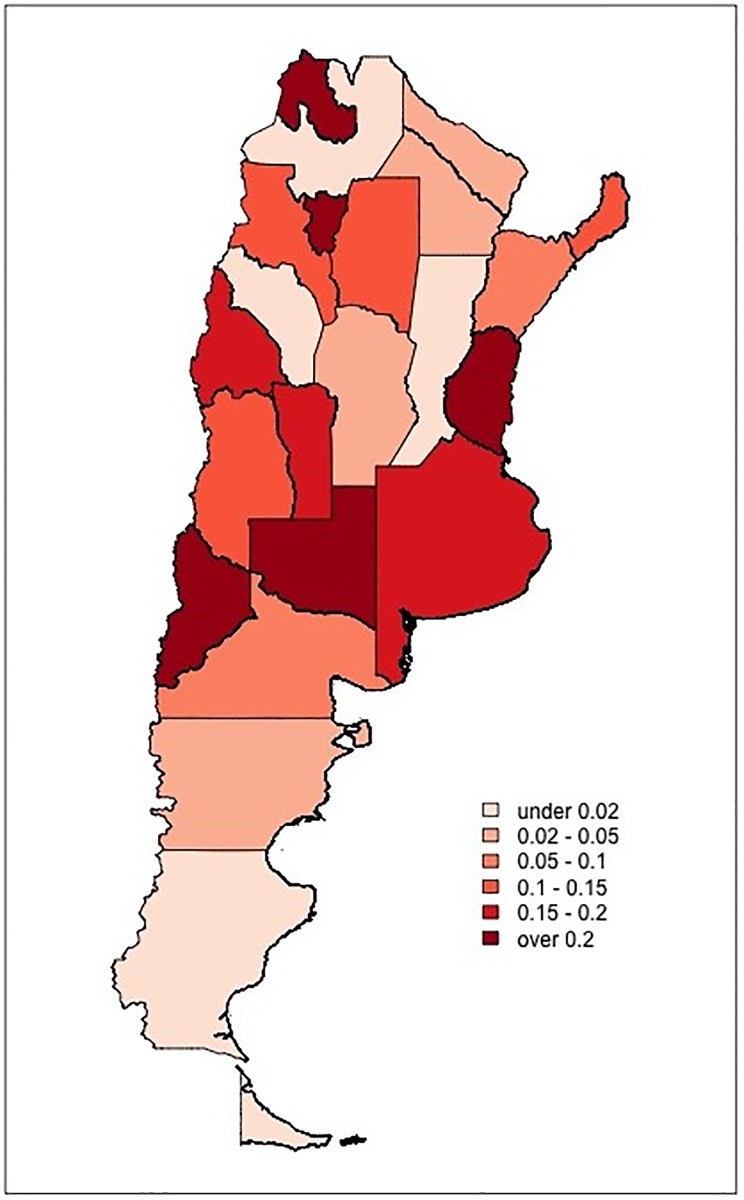
Spatial distribution of BBS risk. The map illustrates the share of voting precincts (*mesas*) classified as at risk of BBS in each Argentinean province. Darker shades correspond to greater risk.

**Fig 5:**
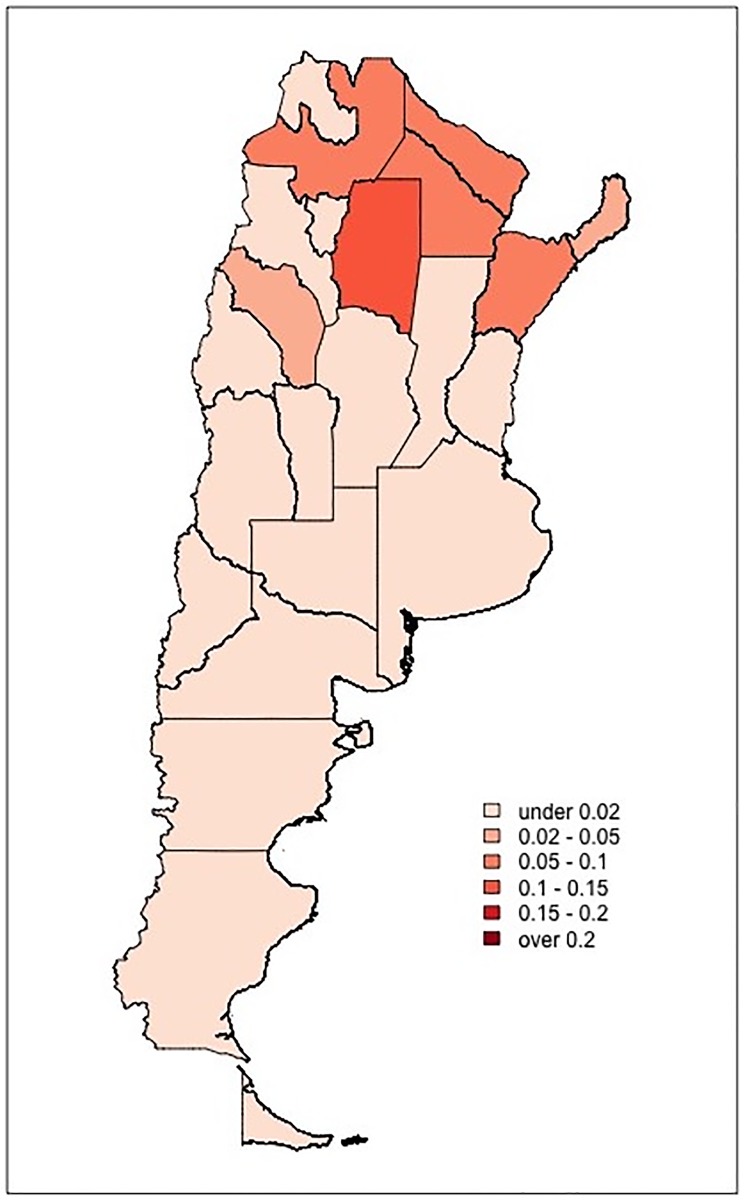
Spatial distribution of VS risk. The map illustrates the share of voting precincts (*mesas*) classified as at risk of VS in each Argentinean province. Darker shades correspond to greater risk.

We next looked at the relationship between predictions and basic demographics of the departments where voting precincts are located. The department-level demographic information was drawn from the 2010 national population census. This analysis suggests that while there is little difference between the aggregate demographics of mesas labeled clean and at risk of BBS, mesas labeled at risk of VS are located in less urbanized departments, with populations experiencing greater need and higher incidence of illiteracy (see Fig A in S1 File). Part of this demographic heterogeneity can be explained by the fact that predicted shares of at-risk *mesas* vary markedly across Argentinean provinces, as provinces differ much in terms of demographic composition. Figs 4 and 5 illustrate the geographic distribution of BBS and VS risk, respectively, over the entire country (for numerical figures, see Table A1 in S1 File). While BBS risk reaches double digits in 10 out of 24 provinces and is relatively frequent in provinces in the central area of the county; VS risk is fairly prevalent in five northern provinces (specifically, in Chaco, Corrientes, Formosa, Salta, and Santiago del Estero) and is otherwise uncommon.

**Fig 6:**
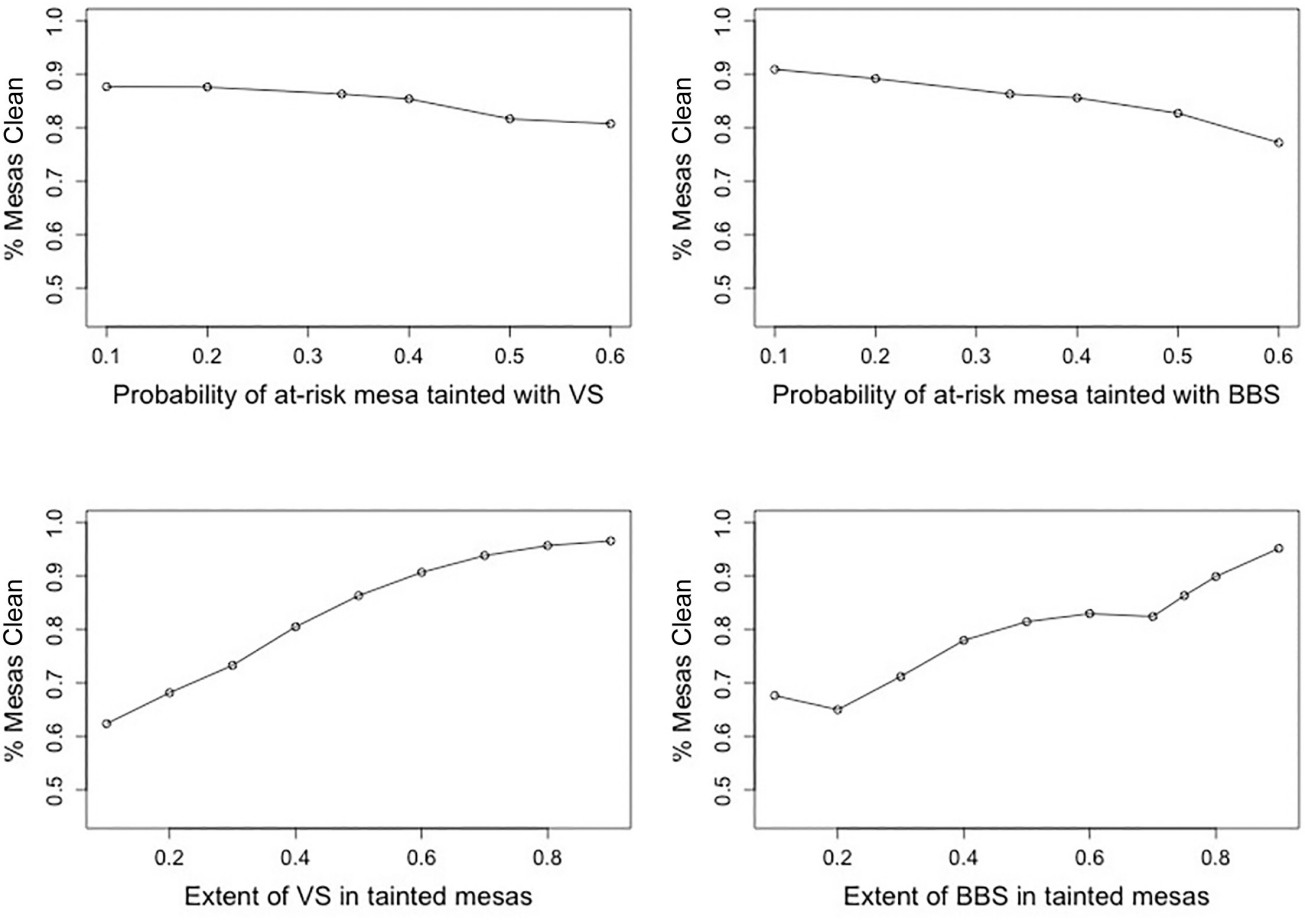
Sensitivity analysis. These figures present how sensitive our predictions of proportion of clean *mesas* are to different parameter values. Top graphs show what happens when the proportion of synthetic *mesas* subject to possible vote stealing or ballot box stuffing varies from 10%, 20%, 33%, 40%, 50% to 60%. The bottom graphs shows what happens when the extent of potential vote stealing or ballot box stuffing in at-risk synthetic *mesas* varies from 10%, 20%, 30%, 40%, 50%, 60%, 70%, 80%, to 90%.

Finally, we evaluate the sensitivity of our predictions to changes in the set of four key parameters used in generating the synthetic training data, holding other parameters constant. Fig 6 shows the results of this sensitivity analysis (for province-level results, see Figs B-E in S1 File). When we increase the probability that any synthetic at-risk *mesa* is subject to VS, the percentage of *mesas* classified as clean remains relatively stable—e.g. 88% labeled clean when only 10% of synthetic at-risk *mesas* are subject to VS, compared to 81% when 60% of synthetic at-risk *mesas* are subject to VS. Predictions are slightly more sensitive to the probability of BBS in the synthetic training data. When only 10% of at-risk *mesas* are affected by BBS, we predict 91% of *mesas* to be clean, compared to 77% when we increase the affected population to 60%. The extent of BBS and VS in tainted synthetic *mesas* has a greater impact on our predictions: when we assume that FPV converts, on average, 10% of other parties’ votes into their own votes in synthetic *mesas* affected by VS, 62% of *mesas* are labeled clean, compared to 97% when FPV converts 90% of competitions’ votes. When we assume that FPV engages in limited BBS (adding only enough ballots to decrease abstention by 10% in synthetic *mesas* affected by this potential fraud), 68% of mesas are labeled as clean; however, when we assume that FPV engages in much more substantial BBS (adding enough ballots to decrease abstention by 90%), the model labels 95% of *mesas* as clean. While this may seem counterintuitive, it is what we expect to see in a simulation like this: when we increase the proportions of at-risk *mesas* subject to VS and BBS, the predicted percentages of clean *mesa* decrease, as we are allowing fewer *mesas* to be clean. When we increase the extent of VS or BBS in tainted synthetic *mesas*, however, only *mesas* with exceedingly large turnout or FPV vote share can be detected by the model as fraudulent, leading to conservative predictions where most *mesas* are labeled clean.

## Conclusion

The approach we use in this paper relies upon synthetic training data to generate precinct-level predictions of fraud risk. Both the use of synthetic data and generation of granular predictions are important contributions of our work. For supervised machine learning tools to be useful for election forensics, the analyst needs training data of some form. It is rare for the analyst to have a list of labeled units (like *mesas* or precincts) to use for forensic purposes, so researchers will need some type of synthetic data. As we discuss above, researchers can use historical demographic and political data, along with substantive or theoretical knowledge about the forms that possible election manipulation or error might take in a particular election, to develop the sort of synthetic training data that we deploy in this paper. These training examples can then be used to identify electoral locations at risk of possible election error or fraud and to estimate the overall extent of potential electoral manipulation, as we illustrated using data from a recent election in Argentina. Clearly, this is an important area for future research; users of forensic methods like these will need reliable guidance about how to develop synthetic training data.

## Supporting information

S1 File Fig A, Basic Demographics by Fraud Risk. Distribution of urbanization, unsatisfied basic needs, and illiteracy, for voting precincts classified as clean, at risk of BBS, and at risk of VS, respectively. Table A, Classification by Province. Proportion of voting precincts classified as clean, at risk of BBS, and at risk of VS, respectively, in each Argentinean province. Fig B, Sensitivity to changes in amount of possible BBS in *mesas* at risk of BBS. Percentage of *mesas* classified as clean when the extent of potential ballot box stuffing within synthetic at risk *mesas* varies between 10% and 90%. Fig C, Sensitivity to changes in probability that *mesas* are at risk of BBS. Predicted percent of *mesas* that are classified as clean when the proportion of synthetic *mesas* subject to potential ballot box stuffing varies between 10% and 90%. Fig D, Sensitivity to changes in amount of potential VS in *mesas* at risk of VS. Percentage of *mesas* classified as clean when the extent of potential vote stealing within synthetic at-risk *mesas* varies between 10% and 90%. Fig E, Sensitivity to changes in probability that *mesas* are possibly at risk of VS. Predicted percent of *mesas* that are classified as clean when the proportion of synthetic *mesas* subject to potential vote stealing varies between 10% and 90%.
(PDF)
