# Supplementary material for: Election forensics: Using machine learning and synthetic data for possible election anomaly detection
Source: PLoS One. 2019 Oct 31;14(10):e0223950. doi: 10.1371/journal.pone.0223950 (PMC6822750; doi:10.1371/journal.pone.0223950)

PLoS One Supporting Information Appendix S1

Article title: Election Forensics: Using Machine Learning and Synthetic Data for Potential Election Anomaly Detection

Authors: Zhang, M., Alvarez, R.M., Levin, I.

The following Supporting Information is available for this article:

S1 Fig. A. Basic Demographics by Fraud Risk. Distribution of urbanization, unsatisfied basic needs, and illiteracy, for voting precincts classified as clean, at risk of BBS, and at risk of VS, respectively.

S1 Table A. Classification by Province. Proportion of voting precincts classified as clean, at risk of BBS, and at risk of VS, respectively, in each Argentinean province.

S1 Fig. B. Sensitivity to changes in amount of possible BBS in mesas at risk of BBS. Percentage of *mesas* classified as clean when the extent of potential ballot box stuffing within synthetic at risk *mesas* varies between 10% and 90%.

S1 Fig. C. Sensitivity to changes in probability that *mesas* **are at risk of BBS.** Predicted percent of *mesas* that are classified as clean when the proportion of synthetic *mesas* subject to potential ballot box stuffing varies between 10% and 90%.

S1 Fig. D. Sensitivity to changes in amount of potential VS in *mesas* **at risk of VS.** Percentage of *mesas* classified as clean when the extent of potential vote stealing within synthetic at-risk *mesas* varies between 10% and 90%.

S1 Fig. E. Sensitivity to changes in probability that *mesas* **are possibly at risk of VS.** Predicted percent of *mesas* that are classified as clean when the proportion of synthetic *mesas* subject to potential vote stealing varies between 10% and 90%.

Basic Demographics by Fraud Risk

Fig. A presents box plots showing the distribution of urbanization, unsatisfied basic needs, and illiteracy, for voting precincts classified as clean, at risk of BBS, and at risk of VS, respectively.

Figure A: **Basic Demographics by Fraud Risk**

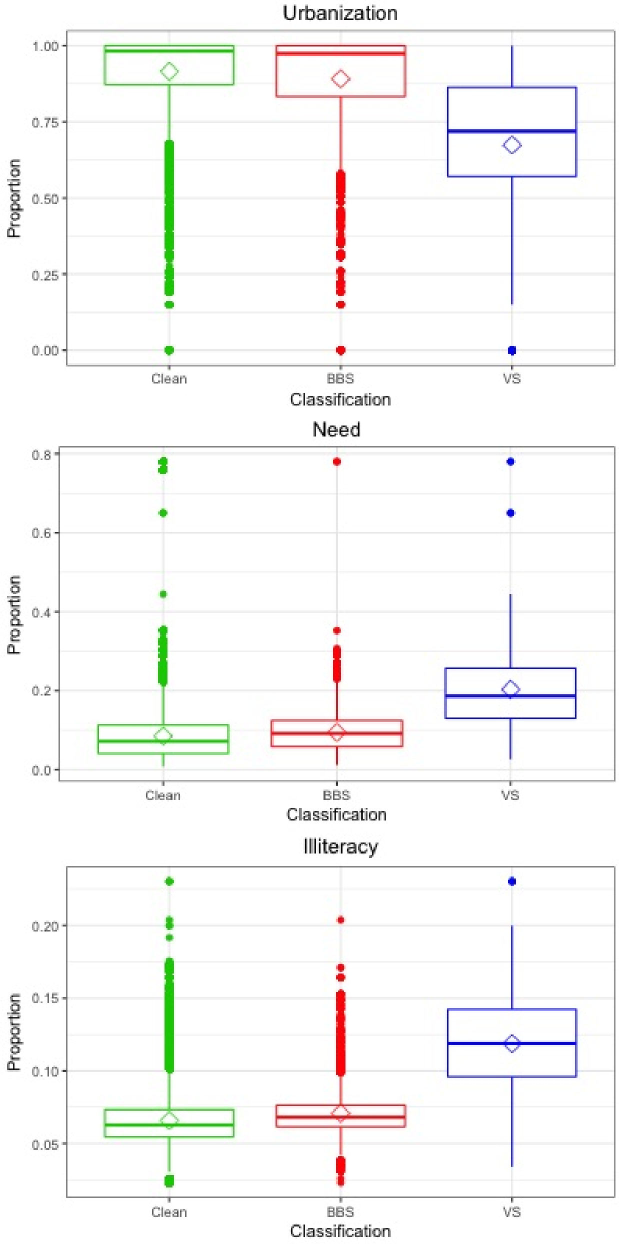

Classification by Province

Table A presents the proportion of voting precincts classified as clean, at risk of BBS, and at risk of VS, respectively, in each Argentinean province.

Table A: **Random Forest Model Performance on Training Data**

	Clean	BBS risk	VS risk
Buenos Aires	81.6	18.2	0.2
Catamarca	88.6	10.6	0.7
Chaco	87.5	2.7	9.9
Chubut	93.8	4.0	2.2
Buenos Aires City	98.0	1.7	0.3
Cordoba	96.9	2.9	0.2
Corrientes	88.3	6.0	5.6
Entre Rios	65.7	34.1	0.2
Formosa	89.8	4.7	5.5
Jujuy	75.4	24.5	0.1
La Pampa	78.5	21.3	0.1
La Rioja	95.0	2.4	2.6
Mendoza	85.8	13.9	0.3
Misiones	82.2	14.7	3.1
Neuquen	69.3	30.0	0.7
Rio Negro	90.0	8.1	1.9
Salta	89.4	1.5	9.1
San Juan	82.2	16.9	0.9
San Luis	84.3	15.5	0.2
Santa Cruz	96.9	1.5	1.6
Santa Fe	97.6	1.7	0.7
Santiago del Estero	77.6	12.2	10.2
Tierra del Fuego	98.6	0.7	0.7
Tucuman	79.3	20.1	0.6

Sensitivity Analyses by Province

In the following figures we provide the same type of sensitivity analyses that are shown in the text in Fig 6. These additional figures provide further insight into the sensitivity of our model's predictions to variation in the level of possible ballot box stuffing or vote stealing. Fig B shows the sensitivity of our model's predictions for the percentage of clean *mesas* in each province when we use different values for the extent of possible ballot box stuffing in the synthetic *mesas* in that province. Similarly, Fig C shows the predicted percent of *mesas* that are clean (vertical axis), when the proportion of synthetic *mesas* at risk of ballot box stuffing increases from 10% to 90%.

Figure B: **Sensitivity to Changes in Amount of Possible BBS in Mesas at Risk of BBS**

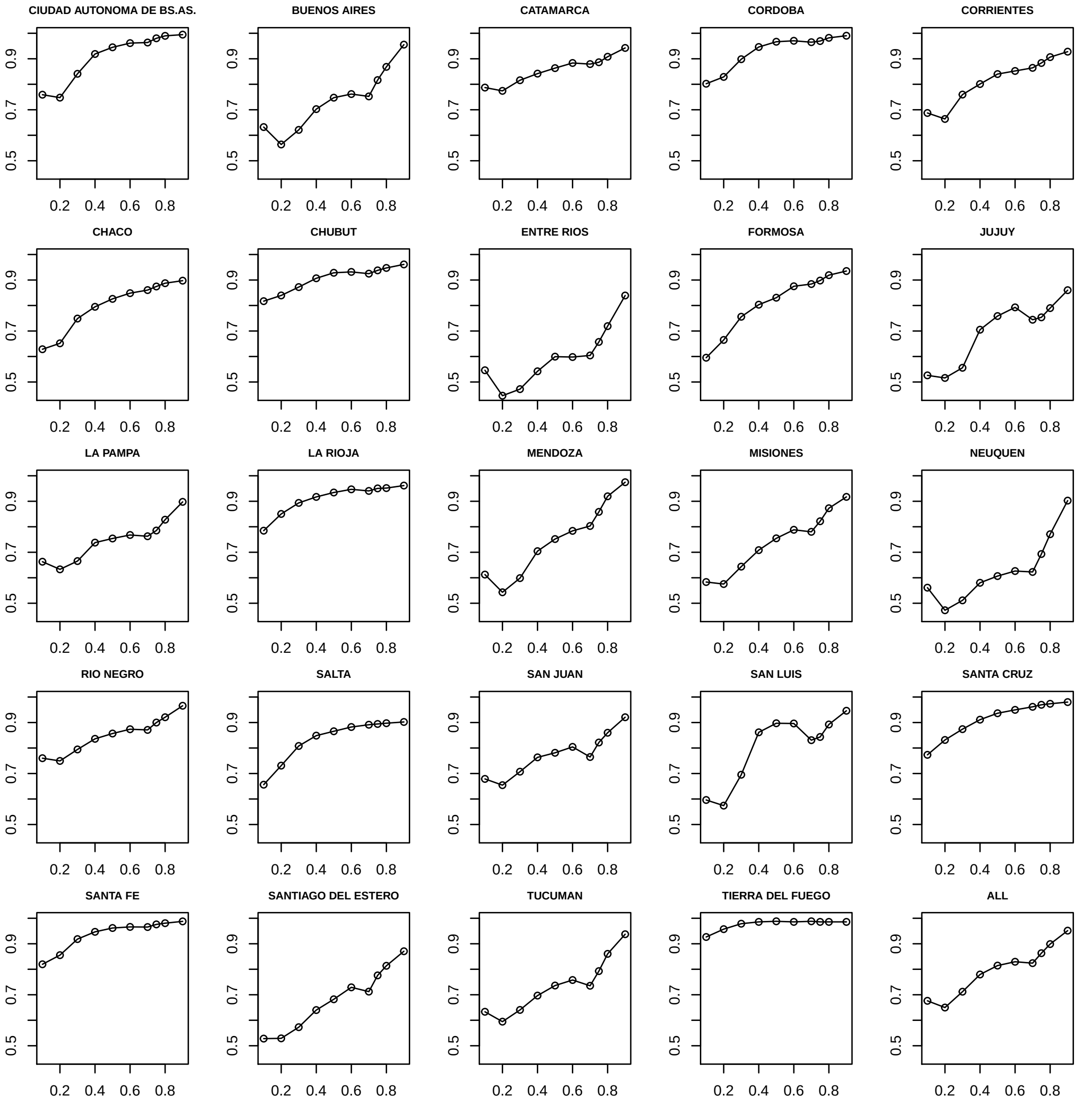

Figure C: **Sensitivity to Changes in Probability That Mesas Are At Risk of BBS**

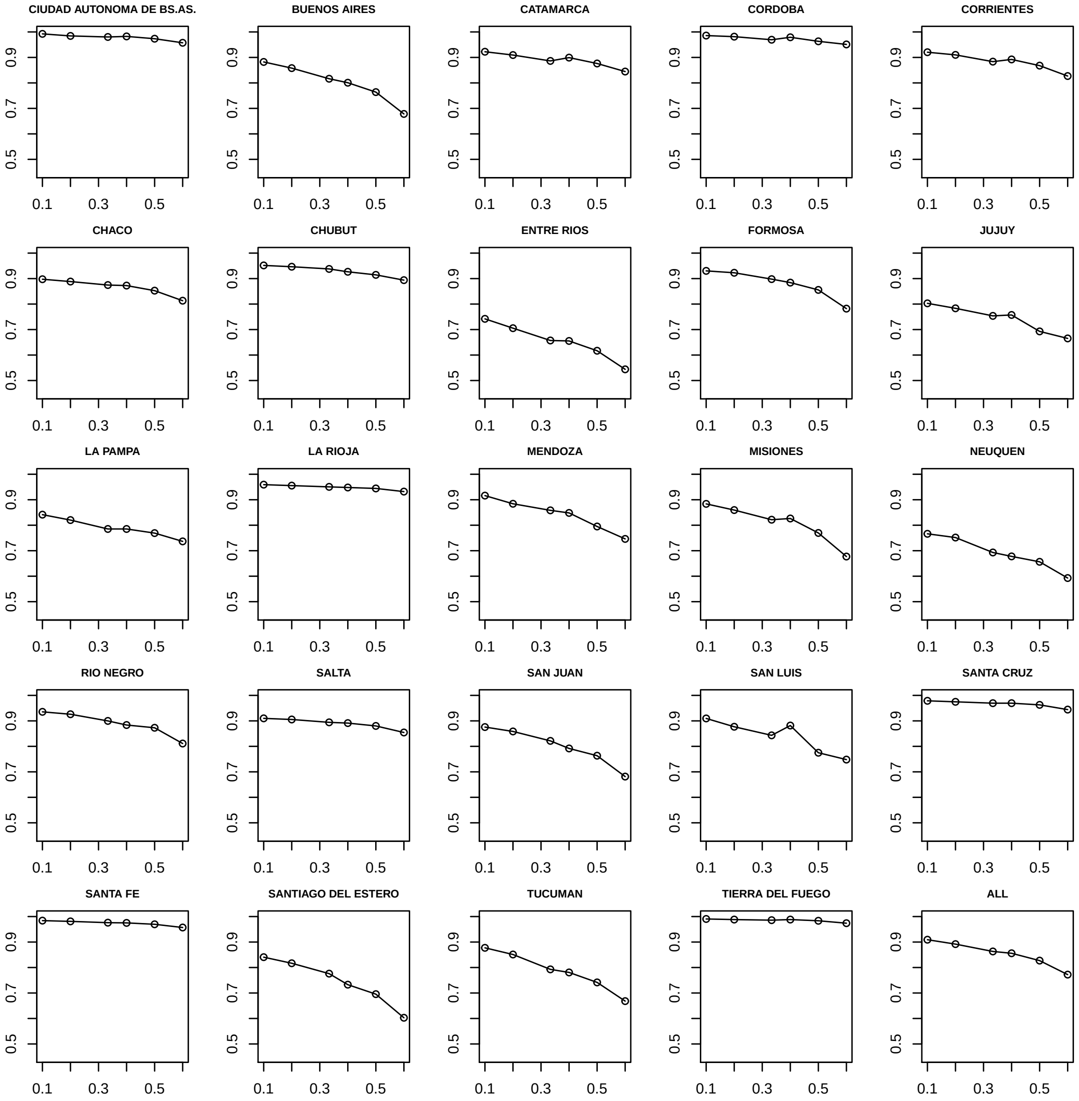

Similarly, Fig D shows the sensitivity of our model's predictions for the percentage of clean *mesas* in each province when we use different values for the extent of possible vote stealing in the synthetic *mesas* in that province. Finally, Fig. E shows the predicted percent of *mesas* that are clean (vertical axis), when the proportion of synthetic *mesas* at risk of possible vote stealing is increased from 10% to 90%.

Figure D: **Sensitivity to Changes in Amount of Potential VS in Mesas At Risk of VS**

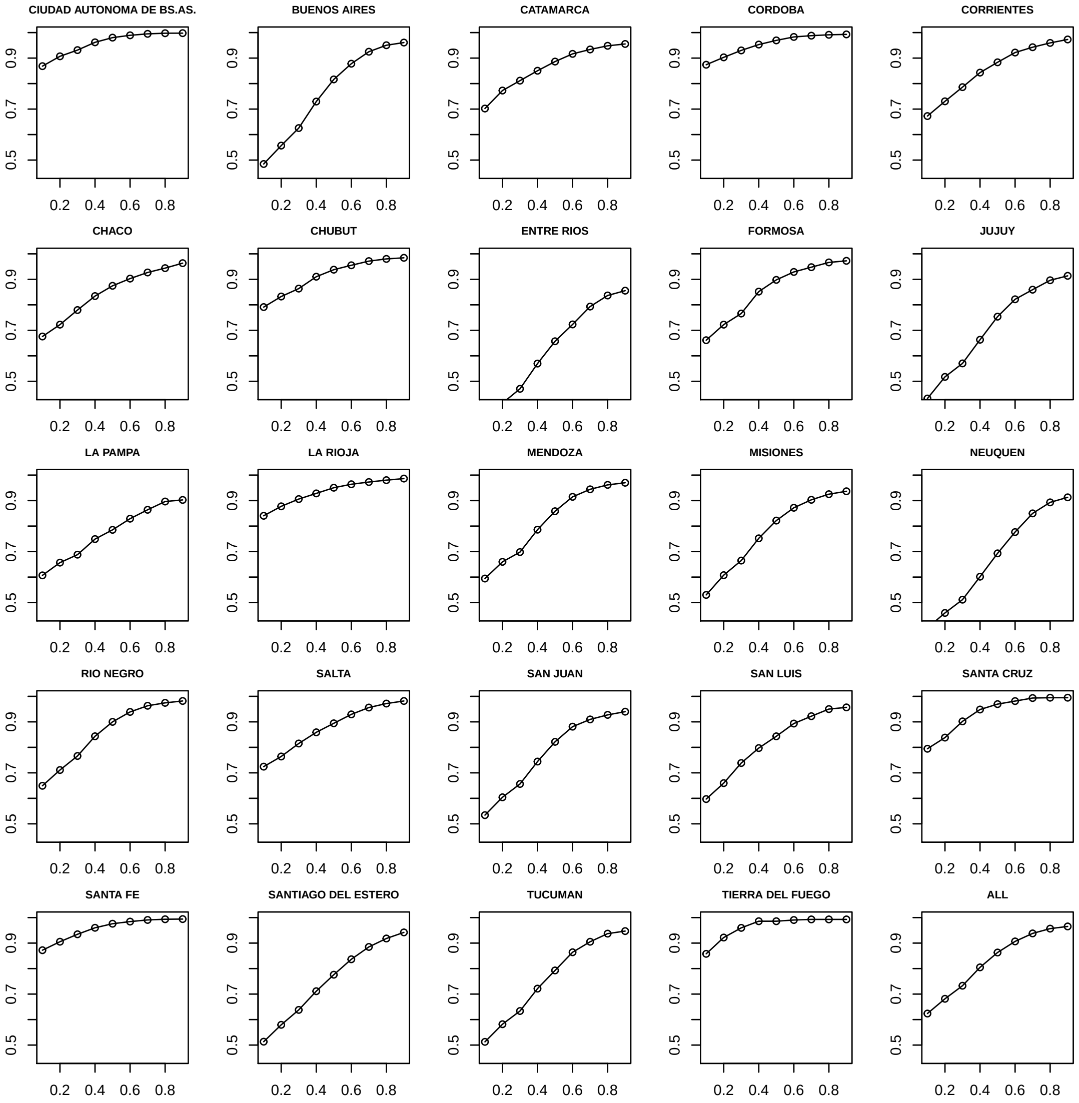

Figure E: **Sensitivity to Changes in Probability that Mesa are Possibly At Risk of VS**

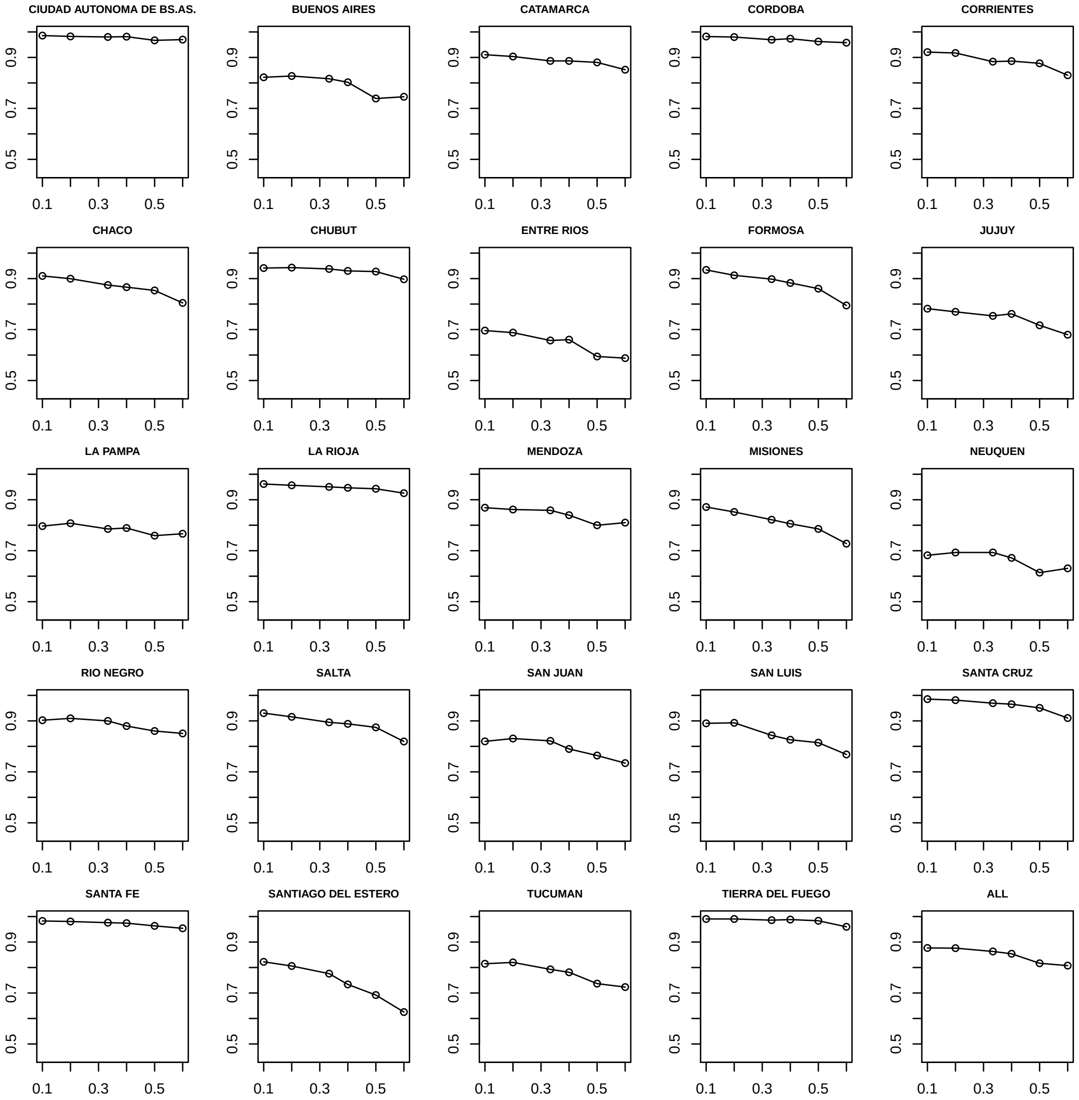

Supplement: S1 File — Fig A, Basic Demographics by Fraud Risk. Distribution of urbanization, unsatisfied basic needs, and illiteracy, for voting precincts classified as clean, at risk of BBS, and at risk of VS, respectively. Table A, Classification by Province. Proportion of voting precincts classified as clean, at risk of BBS, and at risk of VS, respectively, in each Argentinean province. Fig B, Sensitivity to changes in amount of possible BBS in mesas at risk of BBS. Percentage of mesas classified as clean when the extent of potential ballot box stuffing within synthetic at risk mesas varies between 10% and 90%. Fig C, Sensitivity to changes in probability that mesas are at risk of BBS. Predicted percent of mesas that are classified as clean when the proportion of synthetic mesas subject to potential ballot box stuffing varies between 10% and 90%. Fig D, Sensitivity to changes in amount of potential VS in mesas at risk of VS. Percentage of mesas classified as clean when the extent of potential vote stealing within synthetic at-risk mesas varies between 10% and 90%. Fig E, Sensitivity to changes in probability that mesas are possibly at risk of VS. Predicted percent of mesas that are classified as clean when the proportion of synthetic mesas subject to potential vote stealing varies between 10% and 90%. (PDF) [file pone.0223950.s001.pdf]
